# Observations upon Menstruation—Its Cause, Character and Effects upon the Female Economy

**Published:** 1856-09

**Authors:** Robert E. Campbell

**Affiliations:** Benton, Lowndes County, Alabama


### ARTICLE III.

*Observations upon Menstruation—its Cause, Character and Effects upon the Female Economy.* By Robert E. Campbell, M. D., Benton, Lowndes County, Alabama.

Any remarks that I may offer upon this subject, may appear superfluous, after the numerous essays which have been published upon it, yet the surest way to come at a correct conclusion, when a mooted point is in question, is by deep thought and research, neither of which I profess to bring forward in the ensuing remarks; yet, I hope that the facts I may present, will stimulate Southern practitioners to the study of menstruation in our climate. It is indeed a subject well worthy of the attention of medical men, for the affections and ills which spring from its unnatural course, are many and various—the greater part of them constituting the most serious maladies with which we have to contend in our ordinary routine of female practice.

The most approved definitions of *menstruation* say it is "a secretion from the female generative organs—occurring about every twenty-eight days." This is about the sum and substance of them all, and therefore it would be unnecessary for me to quote at length the various definitions of authors. There has been, also, much discussion as to the definition, but we will take the old phrase, simply protesting against the word "secretion," for it is not a "secretion." Secretion is defined to be "an organic function, which is chiefly executed *in the glands, and consists in an elaboration or separation of the materials of the blood* at the very extremities of the arterial system," &c. Now, how can menstruation be called a *secretion?* There is no *elaboration* or *separation* of the materials of the blood; on the contrary, the blood which is discharged during this period, is almost pure; and it "exudes in the same manner from the internal parieties of the uterus, as in hemorrhages from exhalation"—(Condie.) He also says that its indisposition to coagulate results from its admixture with the acid discharges of the vagina. From post mortem examinations of females who have died while the menstrual discharge was on them, it has been proven by the highest authority, that the blood-vessels of the uterus are much distended, and that by pressing them, drops of pure blood can be driven out into the uterine cavity. Therefore, I contend that it is not a *secretion*, but a *function.*

It would be an utter waste of time and space to go into a full detail of menstruation, its course, &c., therefore I shall content myself with hurridly glancing at a few prominent points observable in women who are "regular," and also a few who are "irregular."

First, the period at which the function occurs: this, most authors contend varies in different climates. Now, admitting that climate has some effect upon it, we must, after a careful investigation, concede less influence to it, and more to the habits of life, the society, and the state of civilization in which destiny has thrown the female. In our climate, the females of the higher classes of society seldom or ever menstruate before the 14th or 15th year; and cases are not rare where they are destitute of it to the 19th or 20th. Our slaves scarcely ever menstruate before the 15th year; at least, I found such to be the case in the investigations I have made, and I think I can safely put down their natural menstrual age at 15, (too early venereal indulgence of course excepted,) and instances of their not menstruating until a much later period, are quite as frequent as in the "fairer sex." I hope I will not be understood as trying to bring forward the hypothesis, that climate has *no effect* in producing or retarding the menstrual flux: far from it; my idea is, as I said before, that too much stress is laid upon the influence of climate over the function. Therefore, I shall contend that, of our population, the Anglo Saxon menstruate latest, (say 16 years of age,) the African a little sooner, (about 15,) and the mulatto from 15 to 17, and among the remnant of our Indian tribes, (a race famed for virtue,) in this climate, menstruation is rare before the 16th or 17th year. This, to some, may appear a strange doctrine, yet, of the facts stated above, I can assert that they are the result of a good deal of research, which I have carried on for the last four years upon this subject. I believe that if our white females were let loose, unrestrained, as it were, to gratify their animal passions, as are the greater part of our negroes, especially field hands, that they would menstruate equally early with negroes; yet in the present happy state of society, they do not, *nor do female slaves kept as house servants* (ceteris paribus) *come under this function as soon as field hands.* The only reason I can assign for this, is that the former are less liable to give vent to their venereal passions at so early an age as the latter. As far as my observations go, negroes, as a general thing, *discharge less* at each menstrual period than whites: this is probably owing to the latter leading more indolent lives, and their diet being richer and more plentiful. Whites are also more subject to derangements of this important function; therefore, diseases of the uterus are far more frequent than in negroes—the most common affection among blacks being prolapsus uteri.

Suppressio mensium is far more common in this climate than is generally imagined. I have been credibly informed by a medical gentleman of this State, who has done a large female practice for 15 or 20 years, that it is far more frequent than any other form of disease to which women are liable. He attributes it, and I think with good reason, to the sudden changes of temperature, and that especially during the fall and winter months, not to enumerate the cases of it which are produced by fashionable folly. Negroes, on account, perhaps, of exposure, are more frequently afflicted with it.

*Vicarious Menstruation.*—It is a well authenticated fact, that when the discharge does not occur *per naturalis vias*, that it is apt to seek some other outlet. The system being in a state of plethora, nature designs that it should be relieved, and if any obstruction, whether congenital or otherwise, prevent the evacuation of the overloaded vessels, it takes place from some unusual spot. I know of a case, a negro girl, in a neighboring county, who is now about 17 years of age, of a rather anæmic constitution, in whom menstruation proper has never occurred. Her first "show" was about 12 months ago, when a substance of a glairy mucous character was evacuated to the amount of about 3 ounces. She then missed several "periods" without any discharge, and but little, if any, inconvenience. When the time approached, about four months after the first, she was seized with slight hemorrhage from the nose upon the least exertion: this lasted two days, and on the third there was a very profuse bleeding, (to the amount of 20 ounces, I am informed.) There was a female patient admitted to the wards of the Savannah City Hospital, last summer, who was afflicted with a large indolent ulcer of a syphilitic character, upon the anterior part of the left leg, about midway down the tibia. I was, at that time, house student, and observed the case closely; and I observed that at the return of each menstrual period, the sore became turgid with blood, and there was a continual oozing of blood and pus from it, lasting from two to four days, when it would gradually subside. She also told me that she had not had her menses in several months, and that since their suppression, the sore had never failed to ooze blood and matter at the proper "period." Almost every one who has paid any attention to this interesting subject, can detail cases very similar, and in any or all of our medical works, we find various cases detailed of hemorrhages taking place from various parts of the body to supply the accidental deficiency of nature; thus, we have instances of hemorrhages from the eyes, rectum, nose, gums, and even from the cutaneous surface.

*Theory of Menstruation.*—Innumerable theories have been established to account for this function. Every author has brought forward one of his own, and now the medical annals teem with them, the student is often puzzled to select from among the number the most plausible one, and is consequently left in doubt as to a belief upon the subject. The faculty are divided, and scarcely any two of them arrive at the same conclusion. How important is it that we should discuss, through the pages of our Southern periodicals, this important point; for by that means, we may become more conversant with it, and from the collected experience of our brethren, we may possibly glean some facts which will clear up much of the mystery which now envelopes it.

We know that so soon as menstruation is well established, the female is, as a general thing, capable of procreation. There are some instances of women becoming *enciente* before the age of puberty, yet we must look upon all such cases as anomalies, and in no manner affecting the general rule. At this period, the whole corporeal and mental being undergoes a marked change; the limbs become rounded, and assume that beautiful symmetry so characteristic of the well developed female; girlish sports and pastimes are laid aside for more matronly pursuits, and there is a pensive countenance never observed in them before. But, to enumerate all of these changes, would be useless, for every one is conversant with them. I contend that all women who are well formed and healthy, are capable of becoming impregnated after the first menstrual period. Nature, it seems, has made a wide provision in the female economy whereby, after menstruation is established, she generates more blood than is necessary for the wants of her own system. This superabundance of blood is designed for the support of the product of conception. This seems to be clearly proved by the fact, that *when conception takes place the menstrual drain ceases*, and the blood, instead of becoming a source of irritation, and being thrown off as formerly, now goes to the support of the fœtus, as nature designed. When conception does not take place, the accumulation is each month (or about every 28 days) thrown off. It exudes from the internal parieties of the uterus, and is pure blood mixed with the acid secretions of the uterus and vagina. The foregoing is a synopsis of the theory established, if I mistake not, by Simon, and when we analyze it carefully, it seems far the most plausible one.

For want of time and space, I am compelled, for the present, to close this article. Will some of our medical brethren not give us their opinions upon this absorbing subject, through the pages of the Journal? It is probable that we might glean *some new* facts upon it, and most assuredly it will *detract* nothing from our present knowledge.

